#  Structural and Dynamic Study of the Transmembrane Domain of the Amyloid Precursor Protein 

**Published:** 2011

**Authors:** K.D. Nadezhdin, O.V. Bocharova, E.V. Bocharov, A.S. Arseniev

**Affiliations:** Shemyakin and Ovchinnikov Institute of Bioorganic Chemistry, Russian Academy of Sciences

**Keywords:** Alzheimer’s disease, amyloid precursor protein, transmembrane domain, NMR spectroscopy, spatial structure, dynamics

## Abstract

Alzheimer’s disease affects people all over the world, regardless of nationality, gender or social status. An adequate study of the disease requires essential understanding of the molecular fundamentals of the pathogenesis. The amyloid β-peptide, which forms amyloid plaques in the brain of people with Alzheimer’s disease, is the product of sequential cleavage of a single-span membrane amyloid precursor protein (APP). More than half of the APP mutations found to be associated with familial forms of Alzheimer’s disease are located in its transmembrane domain. The pathogenic mutations presumably affect the structural-dynamic properties of the APP transmembrane domain by changing its conformational stability and/or lateral dimerization. In the present study, the structure and dynamics of the recombinant peptide corresponding to the APP fragment, Gln686-Lys726, which comprises the APP transmembrane domain with an adjacent N-terminal juxtamembrane sequence, were determined in the membrane mimetic environment composed of detergent micelles using NMR spectroscopy. The structure obtained in dodecylphosphocholine micelles consists of two α-helices: a short surface-associated juxtamembrane helix (Lys687-Asp694) and a long transmembrane helix (Gly700-Leu723), both connected via a mobile loop region. A minor bend of the transmembrane α-helix is observed near the paired residues Gly708-Gly709. A cholesterol-binding hydrophobic cavity is apparently formed under the loop region, where the juxtamembrane α-helix comes into contact with the membrane surface near the N-terminus of the transmembrane α-helix.

##  INTRODUCTION 

Over a century ago, the German physician A. Alzheimer described the degenerative brain disease that manifests itself in selective neural degeneration in the regions of the brain cortex responsible for cognitive perception and memory [1]. This disease can be inherited or appear sporadically; the inherited form (the so-called familial forms of Alzheimer’s disease) appears earlier in the life of a subject. Despite the considerable progress achieved in the study of the molecular fundamentals of the pathogenesis of Alzheimer’s disease [2], currently available therapy can only slow down the development of the disease, not cure it. During the progressive stages of the disease, the amyloid β-peptide (Aβ) accumulates at neuron contact sites outside nerve cells and aggregates into ordered bundles or fibrils, forming the so-called amyloid plaques [1]. The presence of hydrophobic protein aggregates leads to failure of nerve impulse transmission [1, 3, 4]. Aβ is a product of the sequential cleavage of the membrane glycoprotein, which is an amyloid precursor protein (APP), by β- and γ-secretases [1, 5]. Aβ was recently shown to possess powerful antimicrobial activity and, possibly, to be a component of innate immunity in the human nervous system [6]. Aβ is naturally produced in small amounts with a length of 38 to 43 amino acids; indeed, the most widespread isoforms have a length of 40 and 42 amino acids [1, 3, 4]. Normally, the ratio Aβ _1–42_ /Aβ _1–40 _ of peptides is low and amounts to 1/9, but with Alzheimer’s disease it increases significantly, leading to the formation of amyloid plaques; the structural changes in Aβ underlie their formation process [1, 3, 4]. At the same time, some experimental data show that oligomeric forms of Аβ (including intracellular) display a potential neurotoxic effect even before the formation of fibrils and plaques [1, 3, 4].

While it dimerizes in plasmolemma, APP has the multidomain structure of an bitopicmembrane protein [7]. More than half of all familiar APP mutations of Alzheimer’s disease fall on its transmembtrane (TM) domain and juxtamembrane regions [8, 9]. It is currently believed that these pathogenic mutations affect the lateral dimerization of the APP in the membrane, changing the conformation of the dimer and/or its stability. This is considered to be a possible cause behind the alternative APP cleavage by γ-secretase in the membrane and the domination of the pathogenic Aβ _1–42 _ form over Aβ _1–40 _ [9–11]. Meanwhile, it has been demonstrated that the APP-TM domain and its juxtamembrane regions specifically interact with the membrane environment, particularly with cholesterol and Cu ^2+^ and Zn ^2+^ cations, which may have an effect on the structural-dynamic properties and APP dimerization [12–15]. Thus, to understand better the molecular fundamentals of the pathogenesis of Alzheimer’s disease, it is necessary not only to determine the spatial structure of Aβ peptides and their aggregates, but also of the APP protein and its TM domain. Despite some progress in structural-dynamic studies of Aβ amyloid peptides, only theoretical spatial models of the APP-TM domain and its mutant pathogenic forms exist to date. In the present study, the spatial structure is solved by heteronuclear NMR spectroscopy in a membrane mimetic environmet, and the dynamics of the APP-TM domain with a juxtamembrane (JM) region, which was incorporated as a monomer into detergent micelles, is described.

##  EXPERIMENTAL 

**Preparation of NMR samples of the APP-TM domain in a membrane mimetic environmet**

The necessary for NMR amount of ^15^ N and  ^13^ С labeled sample of the recombinant peptide APPjmtm, which corresponded to the APP fragment Gln686–Lys726 with the additional N-terminal residues Gly–Ser that remained after the hybrid protein had been cleaved by thrombin, were prepared according to the procedure described in [16]. The sample’s purity was checked using the ^1^ H/ ^15^ N-HSQC spectra of the isotope-labeled APPjmtm dissolved in 500 µl of a 5 : 5 : 1 chloroform–methanol–water mixture with a peptide concentration of 0.3 mM. Based on a screening of the composition of the membrane mimetic environment, detergent dodecylphosphocholine (DPC) micelles were selected for the subsequent structural NMR studies of APPjmtm [16]. Dry APPjmtm and DPC samples were dissolved in a 1 : 1 trifluoroethanol–water mixture, kept for several minutes in an ultrasound bath, lyophilized, and dissolved in a 20 mM acetate buffer (pH 5.0, 5% D _2_ O). In order to prevent bacterial contamination, 0.05 mM NaN _3_ was added to the samples; 1 mM EDTA was also added for phospholipase inhibition. To attain a higher uniformity of the micelle size, several freeze–thaw cycles (heating to 40–45 ^o^ С) were carried out, followed by storage in an ultrasound bath in order to achieve complete transparency of the solution. All samples were prepared on the basis of 0.3–1 mM of APPjmtm in 400 µl of the micelle solution with a peptide–detergent molar ratio of 1 : 70, which ensured that the peptide content was one per micelle. Accordingly, the DPC concentration was higher than the critical micelle concentration (~1 mM). The prepared samples were placed into Shigemi NMR ampoules by means of a glass plunger. The sample’s quality was assessed using the 2D NMR spectra ^1^ H/ ^15^ N-HSQC. The variation of pH and temperature demonstrated that the best NMR spectra of APPjmtm solubilized in DPC micelles (in terms of signal resolution) are obtained at pH 4.3–5.3 and at a temperature of 40–50°C.

**NMR spectroscopy of the APP-TM domain in a membrane-like milieu**

The NMR spectra of APPjmtm solubilized in DPC micelles at pH 4.6 and a temperature of 45°С were obtained on AVANCE III spectrometers (Bruker BioSpin, Rheinstetten, Germany) equipped with cryoprobes, with proton operating frequencies of 600 and 800 MHz. The spectra were processed with TOPSPIN 3.0 software (Bruker BioSpin, Rheinstetten, Germany). The NMR spectra were analyzed using CARA software [17]. To assign the ^1^ H-, ^13^ C-, and  ^15^ N-resonances of the peptide and obtain the structural data (assignment and integration of cross peaks in NOE), we used an asset of two- and three-dimensional spectra: ^1^ H/ ^15^ N-HSQC, ^1^ H/ ^13^ C-HSQC; ^1^ H/ ^13^ C/ ^15^ N-HNCA, ^1^ H/ ^13^ C/ ^15^ N-HN(CO)CA, ^1^ H/ ^15^ N-HNHA, ^1^ H/ ^13^ C/ ^15^ N-HNCO, ^1^ H/ ^15^ N-NOESY-HSQC, ^1^ H/ ^13^ С-TOCSY-HSQC, and ^1^ H/ ^13^ C-NOESY-HSQC [18]. The data on the intramolecular dynamics of the peptide were obtained via an analysis of the ^15^ N-relaxation data. For that purpose, the values of the heteronuclear ^15^ N{ ^1^ H} NOE, longitudinal ( *Т*_1_ ) and transverse ( *Т*_2_ ) relaxation times, and the rotational correlation time (τ _R_ ) for the ^15^ N-labeled APPjmtm sample were measured based on the procedure described in [19]. The values of the times of exchange of the amide protons of the backbone peptide chain for deuterium of the solvent were assessed according to the changes in the signal intensities in the set of ^1^ H/ ^15^ N-HSQC spectra, which had been sequentially accumulated after 1, 2, 4, 6, 8, 10, 14, 18, and 24 h for the pre-lyophilized APPjmtm sample that was dissolved in D _2_ O and incorporated into the micelles. The degree of spatial remoteness of an amino acid residue from the micelle surface and center were determined based on the broadening of the signals from the amide groups of APPjmtm in ^1^ H/ ^15^ N-HSQC spectra, after 5- and 16-doxyl stearine acids (5- and 16-DSAs) were sequentially added as paramagnetic spin labels in ratios of 0.5, 1, and 2 labels per DPC micelle, respectively. The APPjmtm residues, which take part in cholesterol binding, were identified by analyzing the changes in the generalized chemical shifts Δδ _[HN] _ (which were calculated as a square root of the sum of the squared changes in the chemical shifts of the signals ^1^ H (Δδ _1H_ ) and ^15^ N (Δδ _15N_ /5) [13]) of the cross peaks belonging to the amide groups in ^1^ H/ ^15^ N-HSQC spectra; these peaks being induced by the introduction of cholesteryl hemisuccinate (cholesterol analog) on the basis of one hemisuccinate molecule per DPC micelle.

**Calculation of the spatial structure of the APP-TM domain**

**Fig. 1:**
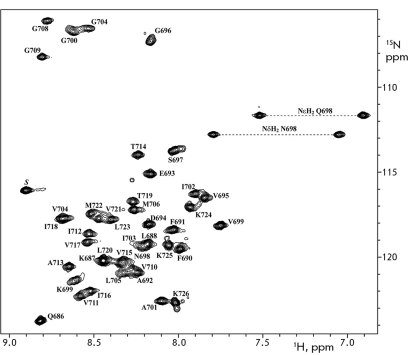
Heteronuclear NMR spectrum ^1^ H/ ^15^ N-HSQC of recombinant uniformly ^13^ C/ ^15^ N-labeled peptide APPjmtm solubilized in an aqueous suspension of DPC micelles with a peptide/detergent ratio of 1:70, рН 4.6, 45 °С. The ^1^ H- ^15^ N side chain and backbone resonance assignments are shown.

The APPjmtm spatial structure was obtained using the standard procedure [18]. The calculation of the spatial structure using NMR spectroscopy data was carried out with CYANA 2.1 [20] software using the method of molecular dynamics in torsion-angle space and the simulated annealing algorithm. The restrictions imposed on the interproton distances that are used during structure calculation were obtained from the volumes of NOE crosspeaks in the ^1^ H/ ^15^ N-NOESY-HSQC and  ^1^ H/ ^13^ C-NOESY-HSQC spectra accumulated with a mixing time * t*_m _ = 80 ms. The restrictions on the dihedral angles were obtained from the values of the ^1^ H, ^15^ N, and  ^13^ C chemical shifts of the NH-, CαH-, and CO-groups of APPjmtm with TALOS software [21]. The restrictions on hydrogen bonds were added after preliminary calculation of the structure, by analyzing the spatial proximity of the amide protons and oxygen atoms of the backbone chain of APPjmtm according to the angle criterion 140° < NHO < 180° and 130° < COH < 170°, and the distance criterion 1.9 Å ≤ d(O, H ^N^ ) ≤ 2.3 Å, 3.0 Å ≤ d(O, N) ≤ 3.4 Å, 3.2 Å ≤ d(C, H ^N^ ) ≤ 3.6 Å [22]. As a result, a set of 100 APPjmtm structures were calculated based on the upper limits for the interproton distances, dihedral angles φ, ψ, and χ ^1^ , hydrogen bonds with consideration of the stereospecific assignment of peptide groups; 20 structures with the minimal target function parameter value were selected as the representative ones. Analysis and visualization of the calculated APPjmtm structures were carried out using CYANA and MOLMOL software [23].

##  RESULTS AND DISCUSSION 

The recombinant peptide APPjmtm containing the APP-TM domain with the adjacent N-terminal juxtamembrane sequence was studied by heteronuclear NMR spectroscopy in a membrane mimetic invironmet - in the form of an aqueous suspension of DPC micelles with a 1 : 70 peptide : detergent molar ratio (which corresponded approximately to one peptide per micelle) at pH 4.6 and a temperature of 45°С. It is of interest that APP undergoes cleavage in cell endosomes at a pH value of approximately 5 [13]. The samples of APPjmtm in DPC micelles remained stable at 45°С for a month, which is acceptable for NMR structural studies. The total amount of cross peaks from the amide groups in the ^1^ H/ ^15^ N-HSQC spectrum ( *Fig. 1* ) coincided with the one estimated based on the primary structure of APPjmtm. This fact points to the absence of a slow (at the NMR scale) conformational exchange, and the content of protein impurities is less than 5%. A standard set of two- and three-dimensional heteronuclear NMR spectra was accumulated to sequentially identify the ^1^ H-, ^13^ C-, and  ^15^ N-resonances of APPjmtm and obtain the structural-dynamic data (ref. the Experimental section).

**Fig. 2:**
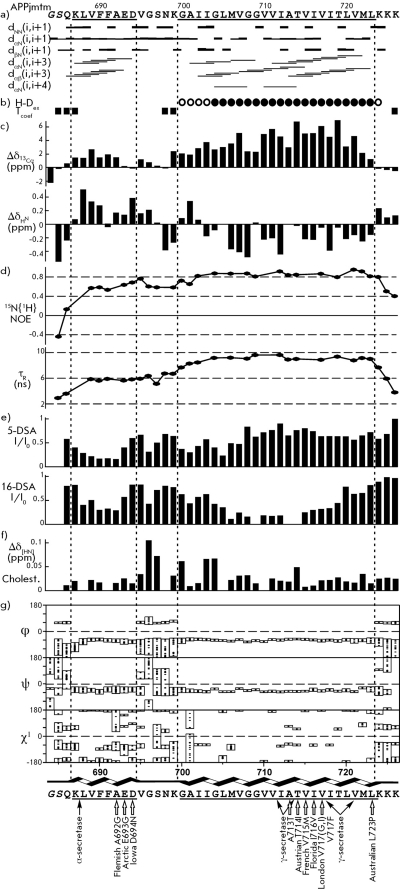
Structural-dynamic NMR data for APPjmtm. *а * - Interproton NOE connectivities observed in the ^1^ H/ ^15^ N-NOESY-HSQC and ^1^ H/ ^13^ C-NOESY-HSQC spectra acquired with 80-ms mixing times. *b * - Water accessibility of the amide groups of APPjmtm solubilized in a DPC micelle aqueous suspension. Slowly hydrogen-deuterium exchanging amide groups of APPjmtm are presented *at the top* according to estimated half-exchange times: t _1/2_ >2 h ( *solid box* ); 1≤t _1/2_ ≤2 h ( *open box* ); for the other residues t _1/2_ <1 h. APPjmtm residues having a temperature dependence of the amide proton chemical shift of more than 3 pbm on 1 °С are marked *at the bottom* by squares, indicating water accessibility to the amide groups. *c * - ^13^ Cα and ^1^ H ^N^ secondary chemical shifts shown *at the top* and *bottom* , respectively, for the APPjmtm residues are given by the difference between the actual chemical shift and typical random-coil chemical shift for a given residue. Pronounced positive or negative Δδ _13Cα_ values indicate a helical structure or an extended conformation (including β-structure) of a protein [18]. The Δδ _HN_ value aside from others strongly depends on the length of a hydrogen bond in which the amide proton participates; thus, the local increase in Δδ _HN_ is specified in the shortening of the given hydrogen bond [24]. *d* - ^15^ N-relaxation data for the APPjmtm amide groups are presented: *at the top* , steady-state ^15^ N{ ^1^ H} NOE; *at the bottom,* effective rotation correlation times * τ *_R_ of Brownian tumbling calculated from a ratio of ^15^ N longitudinal * T *_1_ and transverse * T *_2_ relaxation times. *e* - Amide signal broadening in the ^1^ H/ ^15^ N-HSQC spectra of APPjmtm caused by the addition of 5- and 16-DSA (in a ratio of one spin-label on DPC micelle) tending mainly to be distributed near the surface and the micelle center, respectively. *f* - Variation of the generalized chemical shift Δδ _[HN]_ of amide crosspeaks in the ^1^ H/ ^15^ N-HSQC spectra of APPjmtm caused by the addition of one cholesteryl hemisuccinate molecule in the DPC micelle. *g * - Distribution of backbone and side chain torsion angles φ, ψ and χ ^1^ in a representative set of 20 NMR structures of APPjmtm. APP α- and γ-cleavage sites and mutations associated with familial forms of Alzheimer’s disease [8, 10] are indicated by arrows *at the bottom * of the APPjmtm amino acid sequence.

**Fig. 3:**
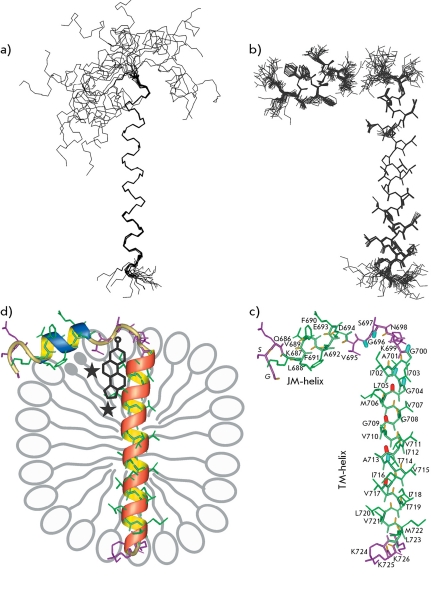
APPjmtm spatial structure. *a * - Set of 20 NMR-derived structures of APPjmtm after superposition of the backbone atoms of the α-helix Gly700-Leu723. Only backbone bonds are shown. *b * - Set of 20 NMR-derived structures of APPjmtm superposed separately on the backbone atoms of α-helices Gly700-Leu723 (JM-helix) and Gly700-Leu723 (TM-helix). Because of uncertainty in the mutual arrangement of the α-helices, the calculated APPjmtm structures are presented with a gap between residues Gly696 and Ser697 from the flexible interhelical loop region. Only heavy atom bonds are shown. *с * - APPjmtm representative spatial structure with APP residue numbering. Heavy atom bonds of helical and unfolded regions are shown in *green* and *violet* , respectively. Amide groups are highlighted in *red* for the residues showing maximum local values of the ^1^ H ^N^ secondary chemical shift (Δδ _HN_ ≈0) along the TM-helix ( *Fig. 2, c* ). Amide groups displaying a significant variation of the [ ^1^ H ^15^ N] generalized chemical shift (Δδ _[HN]_ >0.04) upon addition of the cholesterol analogue to the micelles with embedded APPjmtm (F *ig. 2, f* ) are highlighted in *blue* . Other amide bonds are shown in *yellow* . *d * - Schematic representation of the supramolecular system of the APPjmtm monomer associating with DPC micelle. The JM- and TM-helices of APPjmtm are colored in *blue* and *red* , respectively. Presumable rarefaction of detergent polar heads under the JM-helix are shown with the embedded spin-labels 5- and 16-DSA, the paramagnetic groups of which are depicted by asterisks situated near the surface and centre of the micelle, respectively. The APP cholesterol-binding site apparently formed under the interhelical loop near the N-terminus of the TM-helix is schematically indicated.

It follows from an analysis of the combination of the NMR data obtained that the APPjmtm peptide contains two structured helix regions. The characteristic for the helices *i-* and *i* +3 NOE contacts ( *Fig. 2a* ), positive secondary chemical shifts of the ^13^ Cα signals ( *Fig. 2c* ), and small values of the temperature coefficients of the chemical shifts of ^1^ H ^N ^ signals ( *Fig. 2b* ) are observed here. In the ^1^ H/ ^15^ N-NOESY-HSQC and  ^1^ H/ ^13^ C-NOESY-HSQC spectra, no NOE crosspeaks between the protons of amino acid residues from two helix regions were detected, which likely attests to the absence of interhelix interactions. It was confirmed from a calculation of the spatial structure using the experimental data listed in *Table * that APPjmtm in DPC micelles consists of two α-helices: Lys687–Asp694 and Gly700–Leu723 ( *Fig. 3* ), which are connected via a mobile loop region, Val695-Lys699. The relative orientation of the two helices in the resulting set of APPjmtm structures has not been determined ( *Fig. 3а* ). The structure of each α-helix was calculated with a high level of accuracy ( *Table, Figs. 2g, 3a,b* ). Let us note that the conformation of the backbone and side chains was determined more accurately for the α-helix of Gly700-Leu723.

**Table 1:** Structural characteristics for a representative set of 20 NMR structures of APPjmtm incorporated into DPC micelles

NMR data for structural calculation	Statistics
The total amount of NOE restrictions	318
Intraresidual	111
Interresidual	207
sequential (|i–j|=1)	132
medium range (1<|i–j|<4)	75
long range (|i-j|>4)	0
Restrictions on hydrogen bonds (upper/lower) between the atoms of the backbone chain (24 bonds) between the atoms of the backbone and side chains (0 chains)	72/72 0/0
Restrictions on dihedral angles	74
Angle φ of the backbone chain	30
Angle ψ of the backbone chain	30
Angle χ1 of the side chain	14
Quality of calculation and structural statistics	
Target function of CYANA software (A^2^)	0.38 ± 0.03
Violations of restrictions	
on distances (>0.2 A)	1
on dihedral angles (>5^0^)	0
Pairwise root-mean-square deviation between the structures (A)	
Juxtamembrane α-helix, Lys687–Asp694 residues	
on atoms of the backbone chain	0.23 ± 0.09
on all heavy atoms	1.59 ± 0.28
ТМ α-helix, Gly700–Leu723 residues	
on atoms of the backbone chain	0.14±0.05
on all heavy atoms	0.55±0.10
Analysis of the Ramachandran map (% residues)	
in favorable regions	84.6
in additional, allowed regions	13.5*
in fundamentally allowed regions	1.5*
in forbidden regions	0.4*
* Residues from mobile and nonstructured APPjmtm regions.	

In order to determine the topology of the α-helices of APPjmtm in a DPC micelle, we analyzed the broadening of the signals from the backbone amide protons of a peptide, which was conditioned by their spatial proximity to the paramagnetic spin labels of 5- and 16-DSA, which were mainly distributed near the micelle surface and center, respectively. Based on the pattern of intensity variation in the crosspeaks in the ^1^ H/ ^15^ N-HSQC spectrum after the spin labels were added ( *Fig. 2e* ) and on data on the slow exchange of the protons of amide groups for the deuterium of the solvent ( *Fig. 2b* ), we concluded that the α-helix of Lys687–Asp694 (hereinafter JM-helix) lies in the region of hydrated polar DPC groups, whereas the α-helix of Gly700–Leu723 (hereinafter ТМ-helix) transpierces the hydrophobic part of the micelle. The amphiphility of the short JM-helix also indicates that it is located along the micelle surface, approximately perpendicular to the TM-helix, as shown in *Fig. 3d* . The sequence of weakly polar and hydrophobic amino acid residues at the Gly700–Leu723 region forms an extensive ~40 Å TM-segment. The positively charged amino acids of the side chains Lys699 and Lys724, which flank the TM-helix, presumably interact with the negatively charged phosphate groups of detergent heads. The observed ( *i* +4) periodicity of the secondary chemical shift of the signals from ^1^ H ^N ^ protons ( *Fig. 2c* ) points to the periodic variation of the lengths of the hydrogen bonds H ^N^ ···O ^C ^ along the TM-helix [24]. The amide groups of the residues Leu705, Gly709, Ala713, and Val715 located on one side of the TM-helix ( *Fig. 3c* ) form shorter hydrogen bonds H ^N^ ···O ^C ^ as compared with the residues on the opposite side of the helix. Thus, the TM-helix has a small concavity with a weakly polar surface near the paired residues Gly708–Gly709.

The existence of two independent helix regions in the APPjmtm structure is also confirmed by ^15^ N-relaxation data. The JM- and ТМ-helices of APPjmtm have different characteristic values of the effective correlation time of the rotational correlation time τ _R_ (~6 and ~9 ns, respectively), which are calculated from the *Т*_1_ / *Т*_2_ ratio ( *Fig. 2d* ). The mass of the supramolecular complex estimated from the value <τ _R_ > ≈8 ns averaged over the helix regions, according to empirical dependence [25], is estimated at 27 kDa; this value corresponds to the APPjmtm monomer surrounded by approximately 57 detergent molecules (the typical composition of a DPC micelle [26]). A significant difference (~3 ns) in <τ _R_ > values for residues of the JM- and TM-helices is partially accounted for by the APPjmtm topology with a perpendicular relative arrangement of α-helices in an anisotropically rotating micelle. In addition, APPjmtm rotation inside the micelle and an enhanced lateral mobility of the JM-helix (as compared with the TM-segment) due to the mobile-connecting loop Val695–Lys699 is also possible. A local decrease in the effective correlation time of the rotational correlation time τ _R _ for Ser697 attests to the mobility of the loop region as compared with α-helices. In turn, it follows from the distribution of the values of heteronuclear ^15^ N{ ^1^ H} NOE ( *Fig. 2d* ) that the N-terminus half of the JM-helix and the connecting loop in Val695–Lys699 are mobile in the pico-nanosecond range (0.6≤ ^15^ N{ ^1^ H} NOE ≤ 0.8), unlike the rigid TM-helix ( ^15^ N{ ^1^ H} NOE ≥ 0.8). The increased mobility is responsible for the fact that the conformation of the JM-helix was confirmed less accurately ( *Table, Figs. 2g and 3b* ) and that the structure of the loop region was not determined at all.

The structural-dynamic data obtained in this work corresponds to the results of recent NMR studies [13] of the secondary structure and dynamics of the longer APP fragment – Asp672–Asn770, the so-called C99 peptide, which represents the C-terminus part of APP after it is hydrolyzed by β-secretase. The boundaries of the helix regions of Val689–Asp694 and Asn698–Lys724 and the C99 peptide were determined; they correspond to the α-helices – the short juxtamembrane and long transmembrane ones being connected by a mobile loop. However, the spatial structure of the C99 peptide has not been calculated. Based on the titration data of the C99 peptide in LMPG micelles with a water-soluble cholesterol analog, β-CHOLBIMALT, the ability of APP to function as a cholesterol sensor in the neural membrane was assumed. Indeed, APP is known to accumulate in the human organism in cholesterol-rich microdomains, the so-called lipid rafts [27, 28]. Besides, enzymatic hydrolysis by β-secretase, which results in the formation of β-amyloid, takes place primarily in lipid rafts [28]. It has been also confirmed that non-amyloidogenic hydrolysis by α-secretase occurs outside cholesterol-rich clusters; the enzyme being inactivated as a substrate binds with the lipid rafts [28]. In this work, we studied the binding between APP and a cholesterol analog, cholesteryl hemisuccinate, which incorporates into the micelle. The most significant changes in the generalized chemical shift Δδ _[HN]_ observed near the residues Gly695, Ser696, Gly700, Ile701, Ile703, and Thr713 ( *Fig. 2е* ) point to the site of incorporation of cholesteryl hemisuccinate into the interhelix loop region ( *Figs. 3c,d* ), which agrees closely with earlier obtained data on the specific interaction between the C99 peptide and another cholesterol analog [13].

The data on APPjmtm titration in micelles with paramagnetic spin labels ( *Fig. 2e* ) provides indirect evidence pointing to the fact that there is a hydrophobic cavity near the JM-helix and N-terminus of the TM-helix, which is intended for APP interaction with cholesterol. If the distribution of the 5- or 16-DSA spin labels was uniform, there would have been an equal broadenings of the signals located at the same distance from the DPC micelle center. However, different broadening of the signals of the residues located at different ends of the transmembrane α-helix are recorded in both cases ( *Fig. 2e* ). There are several reasons which could account for this phenomenon. First, the distribution of spin labels over a micelle can be nonuniform due to the specific structure and properties of the APPjmtm incorporated into it. Secondly, the length and size of the side chains in contact with the labels of amino acid residues have an impact on the degree of screening of amide protons (in all likelihood, it is pronounced to the largest extent at G700–Leu705 and Ile718–Leu723 regions). Meanwhile, the considerable broadening of signals at the Leu688–Ala692 region during the titration with the 16-DSA spin label directly points to a local change in the micelle structure under the near-surface juxtamembrane α–helix, which presumably “pushes away” the polar DPC heads (of the lipids), resulting in the formation of a “hydrophobic pocket.” The two spin labels that have small polar groups are incorporated into this “pocket” with some selectivity ( *Fig. 3d* ).

Modification of the ambient conditions and surroundings of APP in the cell membrane may impact the changes in the conformation of the interhelix loop region. This should result in a variation of the mutual orientation of JM- and TM-helices, with a changing size of the cholesterol-binding “hydrophobic” pocket. In turn, it can play a role in simulation of the APP function. The Gly708–Gly709 tandem in the center of the APP–TM helix can, to some extent, compensate for the thickness of the lipid bilayer, which varies depending on the composition of the cell membrane, among other factors; due to cholesterol in the composition of lipid rafts. It is important to note that familial mutations associated with the early progression of Alzheimer’s disease are concentrated not only near the TM-sites of the APP cleavage by γ-secretase, but also at the C-terminus of the JM-helix, where ionogenic Asp693 and Glu694 residues are located ( *Fig. 2* , *bottom* ). Therefore, the determination of the spatial structure of the APP-TM domain with a cholesterol-binding juxtamembrane region in a membrane mimetic environment by heteronuclear NMR spectroscopy is a necessary step in uncovering the molecular mechanism of the alternative APP cleavage, which is associated with the pathogenesis of Alzheimer’s disease.
